# *Bacillus subtilis* Biofertilizer Mitigates N_2_O Emissions from Saline-Alkali Farmland

**DOI:** 10.3390/life16040635

**Published:** 2026-04-09

**Authors:** Rui Li, Xingjie Lin, Yu Miao, Chi Zhang, Fangze Li, Ge Zhang, Qiwei Sun, Tianci Hua, Jiachen Wang

**Affiliations:** 1 BGRIMM Technology Group, Institute Environment Engineering, Beijing 100160, China; xingjielin@sina.com (X.L.); miaoyu_bgrimm@163.com (Y.M.); zhangchiustb@foxmail.com (C.Z.); lfze2016@163.com (F.L.); zhanggling@163.com (G.Z.); b20200050@xs.ustb.edu.cn (Q.S.); 2201110684@stu.pku.edu.cn (T.H.); 2 Research Center for Eco-Environmental Sciences, Chinese Academy of Sciences, Beijing 100085, China; 3 University of Chinese Academy of Sciences, Beijing 100049, China

**Keywords:** nitrogen cycling, soil microbial community, functional gene, nitrification, saline-alkali soil, N_2_O mitigation

## Abstract

Nitrous oxide (N_2_O) emissions from agricultural soils are an important source of greenhouse gases and are strongly influenced by fertilization practices. In this study, a field experiment was conducted from 24 June to 12 October 2024, at a saline-alkali farmland site in Binzhou, Shandong Province, China, to evaluate the effect of Bacillus subtilis biofertilizer on N_2_O emissions and to explore the underlying mechanisms. Compared with conventional chemical fertilization, the *Bacillus subtilis* biofertilizer treatment reduced the cumulative N_2_O emission flux by 39%. At the N_2_O emission peak, the emission flux under the biofertilizer treatment was 40.7%, 18.2% lower than that under the CF and CBF treatments, respectively. Functional gene analysis further showed that at the N_2_O emission peak, the biofertilizer treatment reduced the copy number of *Bacterial*-*amoA* by 94% and 83% relative to CF and CBF, respectively, while the hao gene abundance in the CF treatment was 7.67, 24 times higher than that in the BF and CBF treatments, indicating that the reduction in N_2_O emissions was closely associated with suppression of the nitrification process. In addition, the biofertilizer treatment showed the highest plant nitrogen uptake. All fertilization treatments significantly increased crop yield compared with the control, whereas there was no significant difference in yield among BF, CF, and CBF treatments (*p* > 0.05). These findings indicate that *B. subtilis* biofertilizer can mitigate N_2_O emissions from saline-alkali farmland without reducing crop yield and may represent a promising strategy for sustainable agricultural management.

## 1. Introduction

Nitrous oxide (N_2_O) is a potent greenhouse gas that contributes substantially to climate change and stratospheric ozone depletion. Its atmospheric concentration has increased markedly since the pre-industrial era, largely because of intensified anthropogenic nitrogen inputs, especially those associated with agricultural fertilization [1,2]. Although chemical fertilizers are indispensable for maintaining crop productivity and global food security, their excessive application and the generally low nitrogen use efficiency of agricultural systems have resulted in considerable N_2_O emissions from soils. In addition, fertilizer-derived nitrogen losses can increase nitrate concentrations in groundwater and accelerate eutrophication and harmful algal blooms in aquatic ecosystems [3]. Therefore, improving nitrogen use efficiency while reducing N_2_O emissions has become a key goal for the sustainable development of agriculture.

In China, reducing chemical fertilizer input and adopting more efficient fertilization strategies are considered to be among the most practical approaches for mitigating agricultural N_2_O emissions [4]. In recent years, biofertilizers have attracted increasing attention because they can enhance nutrient availability, improve crop growth, and potentially reduce nitrogen losses. Previous studies have shown that different biofertilizers may reduce soil N_2_O emissions through distinct mechanisms. For example, *Trichoderma viride* biofertilizer reduced N_2_O emissions from fertilized soil by 67.6% [5], whereas *Paenibacillus polymyxa* biofertilizer reduced N_2_O fluxes in tea plantation soils by 36.5–73.1%, mainly through changes in denitrifying microbial communities [6]. In addition, *Bacillus amyloliquefaciens* was reported to reduce N_2_O emissions from potted soil by 50% compared with the control [7]. These findings indicate that biofertilizers can influence N_2_O production, but the responsible microbial pathways may differ among microbial species and soil environments.

Among microbial inoculants used in agriculture, *Bacillus subtilis* has been widely recognized for its strong environmental adaptability, plant growth-promoting capacity, and potential to regulate soil nutrient transformation processes [8]. Compared with many other biofertilizer strains, *B. subtilis* is particularly attractive because of its resilience under stressful soil conditions and its broad application potential in crop production. However, its role in mitigating N_2_O emissions in saline-alkali farmland remains poorly understood, especially from the perspective of nitrification-related functional genes and associated microbial taxa.

Saline-alkali soils provide a particularly important context for studying N_2_O mitigation. High salinity can disrupt nitrogen transformation processes by differentially inhibiting nitrite oxidation and ammonia oxidation, thereby favoring N_2_O production [9]. Salinity is also a major driver of soil microbial community assembly in such systems [10], which in turn affects gaseous nitrogen losses, including NH_3_ and N_2_O emissions. Previous studies have shown that the application of *B. subtilis* biofertilizer can significantly reduce NH_3_ emissions from farmland soils [11]. However, its effects on N_2_O emissions, and the microbial mechanisms underlying those effects, have not yet been clearly determined in saline-alkali agricultural soils.

From a mechanistic perspective, microbial nitrogen transformation pathways are central to soil N_2_O production. In particular, nitrification-related intermediates such as hydroxylamine (NH_2_OH) and nitric oxide (NO) can contribute to N_2_O formation [12]. Moreover, ammonia-oxidizing bacteria may produce N_2_O via nitrifier denitrification, and *Nitrosospira* spp., which are common soil ammonia oxidizers, have been shown to possess this capacity [13]. Therefore, the functional genes *hao* and *norB*, which are associated with hydroxylamine oxidation and NO reduction, may provide important mechanistic insight into how biofertilizer application influences N_2_O emissions. However, previous studies have mainly focused on changes in N_2_O fluxes or denitrifying microbial communities, whereas quantitative analyses of nitrification- and N_2_O-production-related genes such as *hao* and *norB* remain limited. As a result, the contribution of these functional genes to N_2_O mitigation under biofertilizer application is still unclear.

The Yellow River Delta is a typical saline-alkali agricultural region that has experienced persistent salinization under the combined influence of natural processes and human activities [14]. In this region, severe soil salinization, together with intensive fertilizer application, may increase the risk of N_2_O emissions, highlighting the need for improved fertilization strategies that support both crop productivity and environmental protection. We therefore hypothesized that *B. subtilis* biofertilizer could mitigate N_2_O emissions from saline-alkali farmland by regulating nitrogen transformation processes, particularly nitrification-related pathways, and by altering the associated soil microbial community, without reducing crop yield. Accordingly, the objectives of this study were to (1) evaluate the effect of *B. subtilis* biofertilizer on N_2_O emissions from saline-alkali farmland under field conditions; (2) determine its effects on crop yield, nitrogen uptake, and soil physicochemical properties; and (3) explore the underlying mechanisms by quantifying key nitrogen-cycling functional genes and analyzing changes in the soil microbial community.

## 2. Materials and Methods

### 2.1. Experimental Site Description

**Figure 1:**
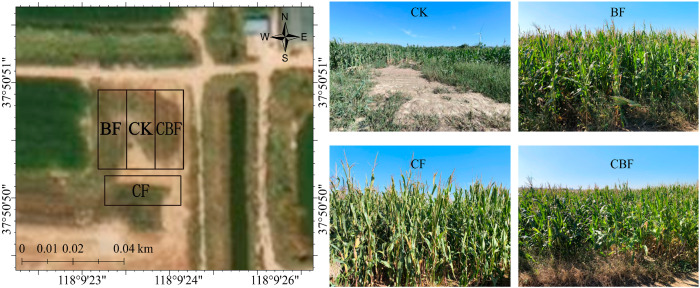
Field test site and different fertilization treatment group schematic diagram.

The field experiment was conducted from 24 June to 12 October 2024, at a planting base in Binzhou, Shandong Province, China (N 39°19′26″, E 116°29′38″) (Figure 1). This region is located in northern Shandong Province, at the terminus of the Yellow River and downstream of the Tuhai River, extending north to the Bohai Sea. It belongs to a warm temperate monsoon climate zone, characterized by distinct continental meteorological features and significant seasonal differences. During the experimental period, the average daily rainfall was 3.02 mm, with an average air temperature of 34 °C and a 0–30 cm soil layer temperature of 27 °C. Rainfall was primarily concentrated in July, with a total precipitation of 223.1 mm. The conventional cropping system in this region involves winter wheat and a summer rotation of maize and soybeans. The experimental area is characterized by severe soil salinization, water shortage, and uneven land distribution, which strongly restrict crop growth. The soil is typical coastal saline-alkali soil with relatively high potassium content and low organic matter. To better describe the initial soil conditions, the main physicochemical properties of the topsoil are summarized in the main text, including pH, electrical conductivity, soil organic matter, total nitrogen, and total soluble salts. In addition, the soil bulk density was 1.35 g·cm^−3^, and the soil texture was classified as loam according to the USDA soil texture classification system. Detailed data are provided in Table S1. The physicochemical properties of the soil in the experimental field are shown in Table S1. The conventional fertilizer applied is diammonium phosphate, with high usage of chemical fertilizers and pesticides, which is a major cause of agricultural non-point source pollution. The biofertilizer was prepared by composting agricultural waste treated with semi-permeable membrane-covered thermophilic compost and inoculating it with the *B. subtilis* [12]. The number of viable *B. subtilis* exceeded 10.0 × 10^8^ CFU·g^−1^, meeting the requirements of China’s NY 884-2012 standard (NY 884-2012; Bio-organic Fertilizer. Ministry of Agriculture and Rural Affairs of the People’s Republic of China: Beijing, China, 2012).

### 2.2. Field Experiment Design

Four treatments were established in this field experiment using a randomized plot design, with five replicates per treatment. Each plot measured 20 m × 8 m. The crop planted during the experiment was Huanong 658 maize (grain maize). The four treatments were as follows: CK, no fertilizer; BF, 100% biofertilizer (13.5 t·ha^−1^); CF, 100% chemical fertilizer (750 kg·ha^−1^); and CBF, 50% chemical fertilizer (375 kg·ha^−1^) combined with 50% biofertilizer (6.75 t·ha^−1^). The actual nitrogen input for all fertilization treatments was 135 kg N·ha^−1^, and all fertilizers were applied as a basal application before sowing, with no additional topdressing. Other field management practices, such as irrigation, weeding, and pest control, were carried out according to local conventional farming practices.

### 2.3. Collection and Detection of N_2_O

The closed static chamber method was used to measure soil N_2_O emission fluxes. The top and sides of the static chamber were wrapped with insulating cotton to minimize temperature fluctuations inside the chamber during sampling. The chamber measured 40 cm × 40 cm × 80 cm (length × width × height) and was inserted 10 cm into the soil during gas sampling. After chamber closure, gas samples were collected at 0, 5, 15, and 30 min using a 50 mL medical plastic syringe equipped with a three-way valve, and the collected gas was immediately transferred into 100 mL sealed gas bags for subsequent analysis. During gas collection, the air temperature inside the chamber was recorded simultaneously. N_2_O samples were analyzed using an Agilent 7890A gas chromatograph (Agilent Technologies, Santa Clara, CA, USA) equipped with a micro-electron capture detector (μECD) and an automatic sampler. The N_2_O concentration was calculated based on standard gas calibration and detector response area provided by the National Institute of Metrology, China. N_2_O fluxes were calculated from the linear change in gas concentration over time and were corrected for chamber volume, sampling area, and chamber temperature during sampling.

### 2.4. Soil Sampling and Measurement

For each plot, 5 subsamples were collected from the 0–20 cm soil layer and thoroughly mixed to form one composite sample per plot at each sampling time. After removing visible plant residues and gravel, each composite sample was divided into two parts: one part was sieved through a 2 mm sieve and stored at 4 °C for physicochemical analyses, and the other was stored at −20 °C for molecular analyses. Soil organic matter (SOM) was determined by the loss-on-ignition method according to Chinese standard HJ 761-2015 (HJ 761-2015; Soil and Sediment—Determination of Volatile Organic Compounds—Headspace Sampling Gas Chromatography. Ministry of Ecology and Environment of the People’s Republic of China: Beijing, China, 2015). Total carbon (TC), total nitrogen (TN), and the carbon-to-nitrogen ratio (C/N) were measured using an elemental analyzer (Vario MAX cube, Elementar, Langenselbold, Germany). Soil electrical conductivity (EC) and pH were determined in a 1:25 (*w*/*v*) soil-water extract according to standard laboratory procedures. The concentrations of ammonium nitrogen (NH_4_^+^-N), nitrate nitrogen (NO_3_^−^-N), and nitrite nitrogen (NO_2_^−^-N) were determined using a continuous flow analyzer. Total soluble salts (TSS) content was determined according to Chinese standard NY 1121.16-2006 (NY 1121.16-2006; Soil Testing—Part 16: Determination of Total Potassium in Soil. Ministry of Agriculture and Rural Affairs of the People’s Republic of China: Beijing, China, 2006.), and soil water content (SWC) was determined by the gravimetric method following HJ 613-2011 (HJ 613-2011; Soil and Sediment—Determination of Total Nitrogen—Alkaline Potassium Persulfate Digestion UV Spectrophotometry. Ministry of Ecology and Environment of the People’s Republic of China: Beijing, China, 2011).

### 2.5. Plant Sampling and Measurement

Crop yield in the experimental plots was measured at harvest. Five plants were evenly harvested from each plot, and the aboveground parts of the plants were divided into fruit and stem sections for weighing to calculate the crop yield. Plant samples were collected, dried to constant weight in a 60 °C oven to calculate moisture content, and ground into fine powder passing through a 100-mesh sieve for physicochemical property analysis. An elemental analyzer (Elementar S-TOC cube, Elementar, Langenselbold, Germany) was used to measure TC, total inorganic carbon (TIC), total organic carbon (TOC), TN, and C/N in the plant samples, combined with plant biomass to calculate nitrogen uptake efficiency. For plant analysis, five plants were evenly harvested from each plot at maturity. Plant nitrogen uptake was calculated as

Nitrogen uptake (kg·ha^−1^) = plant dry biomass (kg·ha^−1^) × plant total nitrogen concentration (%)/100.

### 2.6. Soil Genomic DNA Extraction, Amplification, and Sequencing Analysis

The microbial community’s total genomic DNA was extracted according to the instructions of the E.Z.N.A.^®^ Soil DNA Kit (Omega Bio-tek, Norcross, GA, USA). The upstream primer 338F (5′-ACTCCTACGGGAGGCAGCAG-3′) and downstream primer 806R (5′-GGACTACHVGGGTWTCTAAT-3′) carrying Barcode sequences were used to amplify the 16S rRNA gene’s V3-V4 variable region by PCR [15]. The purified PCR products were then used for library construction using the NEXTFLEX Rapid DNA-Seq Kit. Sequencing was performed on the Illumina PE250 platform (Illumina Inc., San Diego, CA, USA). The raw data was uploaded to the NCBI SRA database (Sequence Number: SRP551647). Quality control of paired-end raw sequencing sequences was performed using fastp software [16], and the sequences were assembled using FLASH software [17]. The data was further processed using the DADA2 sequence denoising method to remove PCR amplification errors or sequencing errors, obtaining the true sequence information, Amplicon Sequence Variant (ASV), in the samples. After quality filtering, denoising, merging, and chimera removal, a total of 993,369 high-quality reads were retained from 39 soil samples, with an average of 25,471 reads per sample (range: 21,452–29,746). For alpha- and beta-diversity analyses, the ASV table was rarefied to 3598 reads per sample, corresponding to the minimum sequencing depth across samples. Taxonomic composition analyses were performed using relative abundances of ASVs. Taxonomic assignment of representative ASVs was conducted using the RDP classifier in QIIME2 (2023.2) against the SILVA 16S rRNA database (v138) with a confidence threshold of 0.7. Differentially abundant taxa were identified using LEfSe (version 1.0) (LDA > 2, *p* < 0.05).

### 2.7. Quantification of Soil Nitrogen Cycle Functional Genes

To explore the potential mechanisms of *B. subtilis* biofertilizer in controlling greenhouse gas emissions, we conducted quantitative analysis of functional genes closely related to N_2_O production in the nitrogen cycle, including Bacterial-amoA, hao, norB, and nosZ. DNA was first extracted from soil samples using a silica-based adsorption column method, followed by quantification in the CFX96 Connect™ Real-Time PCR System (Bio-Rad Laboratories, Hercules, CA, USA). The primers for detecting functional genes are listed in Table S2. Quantification of the functional genes was performed using the SYBR Green method (15 μL reaction system). The reaction program is shown in Table S3. Plasmid standards were diluted in 10-fold gradients to generate the standard curve. Only data with a single effective melting peak and amplification efficiency between 90% and 110% were retained for each primer. Each quantitative PCR assay included two or three no-template controls, with values of zero or negligible amounts for the no-template controls. “Plasmid standards were diluted in 10-fold gradients to generate the standard curves. The amplification efficiency of each primer set ranged from 95% to 105%, and the corresponding R^2^ values were all above 0.99. Only assays with a single melting peak and amplification efficiency between 90% and 110% were retained. To improve normality and homoscedasticity, functional gene copy numbers were log10-transformed prior to statistical analysis.

### 2.8. Statistical Analysis

Soil physicochemical properties and bacterial community diversity indices were compiled using Excel, and graphs were plotted using Origin 2024. Statistical analyses were conducted in R version 4.3.1. Prior to one-way ANOVA, data were checked for normality and homogeneity of variance using the Shapiro–Wilk and Levene’s tests, respectively. When necessary, data were log_10−_ or square-root-transformed to better satisfy ANOVA assumptions. One-way ANOVA followed by Tukey’s post hoc test was then used to test differences among treatments. PLS-PM analysis was conducted using the plspm package in R with 1000 bootstrap resamples. The model fit was evaluated using Goodness of Fit (GOF), *R*^2^, and path coefficient significance [18].

## 3. Results

### 3.1. Soil Physicochemical Properties

**Figure 2:**
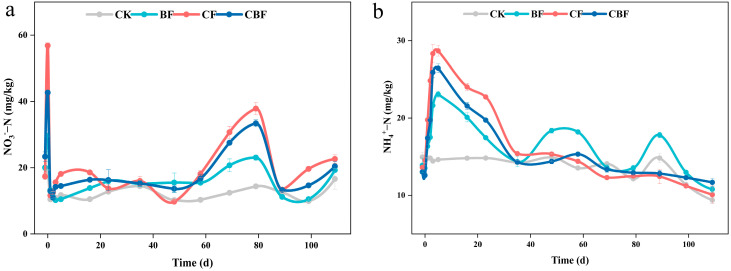
Changes in concentration of NO_3_^−^-N (**a**) and NH_4_^+^-N (**b**) in soil with different fertilization treatments over time. CK (control); BF (100% biofertilizer); CF (100% chemical fertilizer); CBF (50% chemical fertilizer and 50% biofertilizer).

The soil physicochemical properties during the peak period of N_2_O emission are shown in Table 1. The fertilization treatments had no significant effect on soil pH and SWC (*p* > 0.05). Compared to CK, TSS in BF, CF, and CBF were significantly reduced (*p* < 0.05). Compared to CK and CF, SOM and TN in BF and CBF were significantly increased, while C/N was significantly reduced (*p* < 0.05). Both NO_3_^−^-N and NH_4_^+^-N showed rapid post-fertilization increases, followed by clear temporal fluctuations during the observation period. NO_3_^−^-N was largely leached due to irrigation and heavy rainfall in July, but it gradually increased again in mid-August (Figure 2a). NH_4_^+^-N reached its peak on the fourth day after fertilization. Except for CK, BF had the lowest content, and CF had 24.2% higher content than BF (Figure 2b).

### 3.2. N_2_O Emission Flux

**Figure 3:**
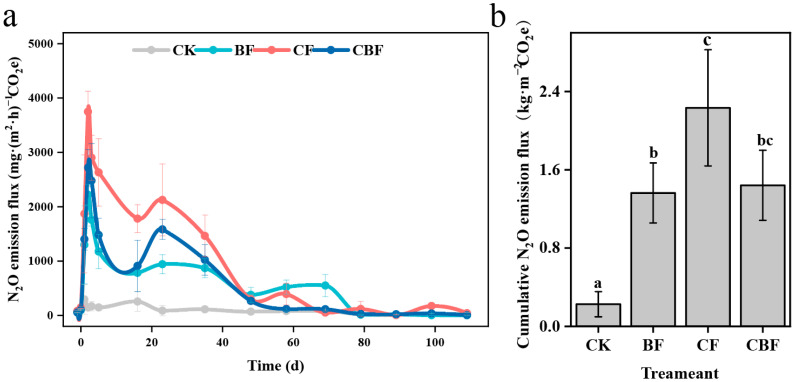
Temporal variation in soil N_2_O flux (**a**) and cumulative N_2_O emissions over the growing season (**b**) under different fertilization treatments. CK, control; BF, 100% biofertilizer; CF, 100% chemical fertilizer; CBF, 50% chemical fertilizer + 50% biofertilizer. Different letters indicate significant differences among treatments (*p* < 0.05).

Throughout the entire growing season of the crops, the emission flux trend for all treatments, except for CK, was similar (Figure 3a). Within one week after fertilization, the N_2_O emission flux rapidly increased, peaking on the third day after fertilization, then gradually decreased, eventually reaching the CK level. N_2_O emissions were primarily concentrated in July. During the peak of N_2_O emissions, BF exhibited the smallest emission flux, 40.7% and 18.2% lower than CF and CBF, respectively, except for CK. The cumulative N_2_O emission flux throughout the experimental period is shown in Figure 3b, with CF having the highest cumulative emission flux, followed by CBF, BF, and CK.

**Figure 4:**
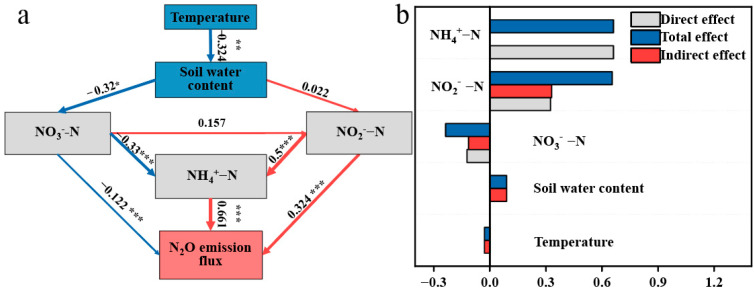
The PLS-PM model of environmental factors and N_2_O emission flux (**a**); the direct and indirect effects of various environmental factors on N_2_O emission flux (**b**). The numbers beside the arrows represent path coefficients, with blue arrows indicating negative effects and red arrows indicating positive effects. The legend/caption of Figure 4b was revised so that the color corresponding to the third bar from the top is now consistent with the figure. The magnitude of the path coefficients is also represented by the thickness of the arrows. Path coefficients were generated after 1000 bootstrap resampling iterations. Significance levels are denoted as * (*p* < 0.05), ** (*p* < 0.01), and *** (*p* < 0.001).

The relationship between environmental factors and N_2_O emission flux was evaluated using Partial Least Squares Path Modeling (PLS-PM). The model results found that NH_4_^+^-N was the most significant factor affecting N_2_O emission flux (r = 0.661), followed by NO_2_^−^-N (r = 0.324), while NO_3_^−^-N showed a negative correlation with N_2_O emission flux (r = −0.122) (Figure 4).

### 3.3. Crop Yield and Nitrogen Utilization

**Figure 5:**
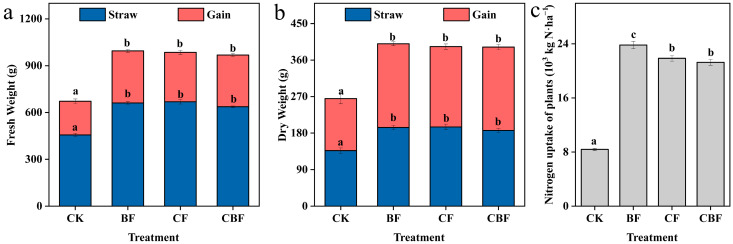
Fresh weight, dry weight, and nitrogen content of grain and straw under different fertilization treatments. (**a**) Fresh weight of straw and grain; (**b**) Dry weight of straw and grain; (**c**) Nitrogen uptake of plants. CK, control; BF, 100% biofertilizer; CF, 100% chemical fertilizer; CBF, 50% chemical fertilizer and 50% biofertilizer. Different letters indicate significant differences among treatments (*p* < 0.05).

The fresh weight, dry weight, and nitrogen content of maize and straw were measured after harvest, and the results are shown in Figure 5. All fertilization treatments significantly increased crop yield compared with CK, whereas no significant difference was observed among the BF, CF, and CBF treatments (*p* > 0.05). The sample size for each treatment and the corresponding ANOVA results are now provided in the Supplementary Materials. The nitrogen uptake by crops is shown in Figure 5c, where the BF treatment had the highest nitrogen content, 2.84 times that of CK, and increased by 8.96% and 12% compared to CF and BF, respectively.

### 3.4. Abundance of Nitrogen Cycling Functional Genes

**Figure 6:**
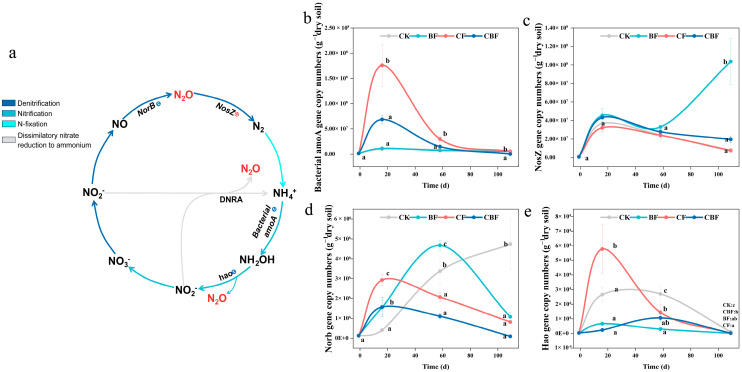
Schematic diagram of nitrogen cycling related to N_2_O production (**a**), Bacterial-amoA (**b**), nosZ (**c**) norB (**d**), hao (**e**). CK (control); BF (100% biofertilizer); CF (100% chemical fertilizer); CBF (50% chemical fertilizer and 50% biofertilizer). Different letters indicate that there are significant differences (*p* < 0.05).

The nitrogen cycle functional genes related to N_2_O production in the soil are shown in Figure 6a. At the peak of N_2_O emission, BF treatment reduced the *Bacterial-amoA* gene copy number by 94% and 83% compared to CF and CBF, respectively (Figure 6b). For the *nosZ* gene, only at the end of the experiment did BF show a significantly higher result than the other treatments, with no significant differences between treatments at other times (Figure 6c). At the peak of N_2_O emission, the *norB* gene copy number in the CF treatment was significantly higher than in other treatments, with CK being the lowest. Subsequently, the *norB* gene copy number in BF treatment gradually increased, reaching its peak at the period of N_2_O emission (Figure 6d). At the peak of N_2_O emission, the *hao* gene copy number in CF treatment was the highest, being 1.17, 7.67, and 24 times higher than CK, BF, and CBF, respectively, and then gradually decreased to the initial level.

Linear regression analysis was used to assess the relationship between nitrogen cycle functional genes and N_2_O emission flux at the peak of N_2_O emission (Figure S1). During the N_2_O emission peak, the BF treatment showed lower copy numbers of *Bacterial-amoA*, *hao*, and *norB* than the CF treatment, indicating a suppression of nitrification-related and N_2_O-production-related processes under biofertilizer application. The pattern of *nosZ* abundance was described according to the figure results, and its ecological implication was interpreted cautiously because *nosZ* is associated with N_2_O reduction rather than production.

### 3.5. Soil Microbial Community

This study conducted high-throughput sequencing and analysis on 39 soil samples from four periods, focusing on the microbial genera related to N_2_O emissions in nitrogen cycling processes. In the nitrification process, fertilization significantly increased the abundance of *Nitrospira* in the soil, with the abundance of *Nitrospira* in the BF treatment being the highest, at 2.18 and 1.53 times that of CF and CBF, respectively. As the amount of biofertilizer increased, the abundance of *Nitrospira* also gradually increased. In contrast, the abundance of *Nitrosospira* was highest in the CF treatment, followed by CBF, showing an increase with the higher application of chemical fertilizers. The alpha-diversity indices were statistically compared among treatments at each sampling stage, and the significance results are now reported in the revised manuscript/figure caption. The abundance of *Nitrosomonas* and *Massilia* showed no significant differences between the treatments. For the denitrification process, fertilization treatments significantly reduced the abundance of *Flavobacterium* and *Pseudomonas*. The CF treatment significantly increased the abundance of *Pedobacter*, while the abundance of *Litorilinea* showed no significant differences between treatments. Additionally, fertilization treatments significantly increased the abundance of *Anaeromyxobacter*, which may indicate an enhancement of the dissimilatory nitrate reduction to ammonium (DNRA) process.

**Figure 7:**
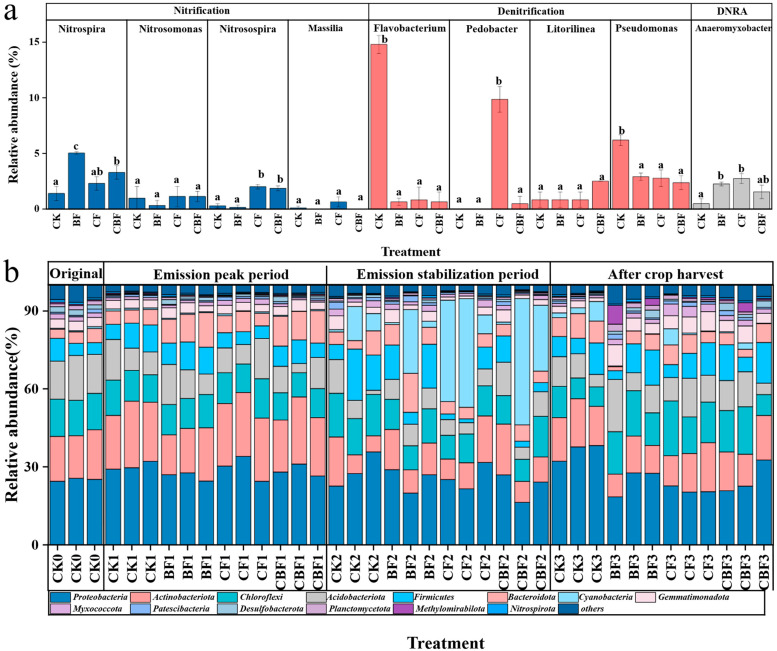
Genera involved in nitrogen cycling processes related to N_2_O emissions (**a**); Bacteria community composition at the phylum level, including 4 periods, respectively: one day before fertilization, 11 July (peak period of N_2_O emission), 22 August (stable period of N_2_O emission), and 12 October (after harvest) (**b**). CK (control); BF (100% biofertilizer); CF (100% chemical fertilizer); CBF (50% chemical fertilizer and 50% biofertilizer). Different letters indicate that there are significant differences (*p* < 0.05) on the third day after fertilization.

The diversity of the soil microbial community is shown in Table 2. In the first two months after maize planting, the richness (sobs, ace, and chao) of the CBF treatment was the highest, while after crop harvest, the BF treatment exhibited the highest richness. At the phylum level, the bacterial community composition (Figure 7) showed that Proteobacteria was the dominant phylum in most periods. During the stable N_2_O emission period, the relative abundance of *Cyanobacteria* was significantly higher in the two fertilized treatments, CF and CBF, even surpassing *Proteobacteria* to become the new dominant phylum. During the peak of N_2_O emissions, the relative abundance of *Actinobacteriota* in the BF group was lower than that in other treatments, while the relative abundance of Acidobacteriota and Firmicutes was higher. LEfSe analysis was used to identify species with significant differences at all stages (LDA ≥ 2, *p* < 0.05). As shown in Figure S2, a total of 90 bacterial genera exhibited significant differences in relative abundance throughout the growing season. Among them, 23, 24, 29, and 14 genera were enriched in CK, BF, CF, and CBF, respectively. Additionally, Oceanobacillus, a potential organic pollutant-degrading genus, was enriched in BF, while Thalassobacillus, a halophilic genus, was enriched in CBF. Some potential pathogens, such as Flavobacterium, were enriched in CK.

## 4. Discussion

### 4.1. Effects of B. subtilis Biofertilizer on Soil Properties and Crop Yield

In the short term after fertilization, the regulation of soil by fertilization is mainly achieved through the impact on soil nutrients and carbon–nitrogen cycling, with no significant effect on pH and SWC. Among them, the overall TSS content in the soil showed a downward trend, mainly due to the irrigation events after fertilization. The dynamic changes in NO_3_^−^-N and NH_4_^+^-N revealed the short-term response characteristics of fertilization on soil nitrogen transformation. In particular, the NH_4_^+^-N content in the BF treatment was significantly lower than in the CF treatment, while the higher nitrogen content in the CF treatment could lead to a higher risk of nitrogen loss in the short term, including ammonia volatilization and nitrogen leaching. In terms of crop yield, all fertilization treatments significantly increased corn yield, with the BF treatment showing the highest nitrogen absorption. This indicates that *B. subtilis* biofertilizer not only meets the normal growth requirements of crops but may also reduce nitrogen loss by promoting efficient nitrogen use [19]. The lower NH_4_^+^-N content observed in the BF treatment may be explained by several complementary processes. First, compared with chemical fertilizer, biofertilizer likely released available nitrogen more gradually, thereby avoiding a sharp short-term accumulation of NH_4_^+^-N after fertilization. Second, the introduced and stimulated microbial community may have enhanced microbial assimilation/immobilization of mineral nitrogen, temporarily retaining nitrogen in the biological pool. Third, the BF treatment showed higher plant nitrogen uptake, indicating that a greater proportion of available nitrogen was absorbed by crops rather than remaining in soil as NH_4_^+^-N. Together, these results suggest that *B. subtilis* biofertilizer may improve nitrogen retention and utilization efficiency under saline-alkali conditions.

### 4.2. The Effect of B. subtilis Biofertilizer on N_2_O Emissions Flux and Its Environmental Influencing Factors

This study demonstrated that *B. subtilis* biofertilizer can mitigate N_2_O emissions from saline-alkali farmland under field conditions. Compared with conventional chemical fertilization, the BF treatment reduced cumulative N_2_O emissions over the growing season by 39% while maintaining crop yield. Among the measured environmental variables, NH_4_^+^-N showed the strongest positive association with N_2_O flux, suggesting that short-term NH_4_^+^-N accumulation after fertilization was an important driver of N_2_O emission dynamics in this system. In contrast, the lower NH_4_^+^-N content under the BF treatment was consistent with its lower N_2_O flux, supporting the interpretation that biofertilizer mitigated N_2_O emissions at least partly by suppressing nitrification-related N turnover [20]. In this study, PLS-PM analysis indicated that NH_4_^+^-N showed the strongest association with N_2_O flux among the measured environmental variables, suggesting that NH_4_^+^-N may be an important factor linked to N_2_O emission dynamics under the present experimental conditions. This may be due to the rapid increase in NH_4_^+^-N in the soil following fertilization, which further promotes N_2_O emissions flux through nitrification [21]. The slow-release nature of the biofertilizer in nutrient release shows its unique advantage. The N_2_O emissions flux showed two fluctuations at 22 August and 2 September, which we speculate are also related to the increase in NH_4_^+^-N content. Furthermore, NO_3_^−^-N content is negatively correlated with N_2_O emissions flux, which may be due to heavy rainfall during the peak N_2_O emissions period, causing NO_3_^−^-N leaching, followed by a gradual increase in NO_3_^−^-N content after the peak, contrary to the overall trend of N_2_O emissions flux [18]. The effect of SWC on N_2_O emissions flux is primarily through promoting the increase in soil NO_2_^−^-N content, which may indicate that the increase in SWC enhances the soil denitrification process, thereby promoting the reduction of NO_3_^−^-N to NO_2_^−^-N [22]. Temperature can affect soil organic carbon balance, moisture content, enzyme activity, and ammonia oxidizer activity, all of which may alter soil N_2_O emissions [23]. Huang et al. found that when the monthly average precipitation reached 78.8 mm, temperature had a greater effect on N_2_O emissions than precipitation. However, in this study, temperature had the least effect on N_2_O emissions flux, indicating that the impact of temperature on soil N_2_O emissions still requires further research. In saline-alkali farmland, high pH and salt stress may further influence microbial physiology by altering osmotic balance, enzyme activity, substrate availability, and the relative competitiveness of ammonia oxidizers and denitrifiers. These factors may modify both the rate and pathway of N transformation, thereby contributing to treatment-specific differences in N_2_O emission dynamics.

### 4.3. Mechanism of B. subtilis Biofertilizer in Reducing N_2_O Emissions Flux

This study explored the mechanism by which *B. subtilis* biofertilizer reduces N_2_O emission flux from two levels. The first is nitrogen cycling functional genes directly related to N_2_O production and reduction. N_2_O emission is closely related to the ammonia oxidation process in the soil, where NH_2_OH produces NO under the catalysis of HAO, and NO is further reduced to N_2_O by an unknown enzyme [12]. Previous studies have shown that known AOA (ammonia-oxidizing archaea) do not encode HAO, and the archaeal enzyme responsible for the hydroxylamine oxidation reaction is still unknown. Additionally, the process of NO being converted to N_2_O via NOR appears to be unique to ammonia-oxidizing bacteria (AOB), as no homologous genes of nor have been found in any AOA or Comammox bacteria [24]. N_2_O production during nitrification is closely related not only to ammonia oxidation but also to nitrifier denitrification. In ammonia-oxidizing bacteria, NH_2_OH can be converted to NO through hydroxylamine oxidation, and NO can then be further reduced to N_2_O through the NOR-related pathway. Previous studies have shown that this pathway is particularly important in AOB, whereas homologous nor genes are absent in known AOA and comammox bacteria. Therefore, the observed reduction in norB abundance under the BF treatment provides additional support for the interpretation that biofertilizer suppressed AOB-associated N_2_O production during the emission peak. Therefore, the impact of AOB on N_2_O emission flux is crucial in known biological processes. In this study, the extreme positive correlation between Bacterial-amoA and hao functional genes and N_2_O emission flux further supports this conclusion. During the N_2_O emission peak, compared to biofertilizer, the use of chemical fertilizer significantly increased the copy numbers of Bacterial-amoA and hao functional genes, thereby promoting the N_2_O emission produced by the nitrification process. The NOR encoded by the norB gene can catalyze the oxidation of NH_2_OH and reduction of NO_2_^−^-N, which is an important source of soil N_2_O. During the N_2_O emission peak, the copy numbers of norB in the CF group were significantly higher than those in other treatment groups, which may also be an important reason for the higher emissions in the chemical fertilizer treatment during the peak period compared to the biofertilizer treatment. By contrast, the pattern of nosZ did not fully parallel the trends of N_2_O flux or N_2_O-production-related genes. This may reflect the fact that nosZ is associated with N_2_O reduction rather than production, and its ecological effect may show a temporal lag relative to short-term N_2_O emission peaks. Therefore, under the present field conditions, the mitigation effect of BF appeared to be more closely associated with the suppression of nitrification-related N_2_O production than with immediate enhancement of N_2_O reduction [25]. In this study, although the nosZ gene copy number in the BF group after crop harvest was 13.5 and 5.29 times higher than in the CF and CBF groups, respectively, no significant difference was observed between treatments during the N_2_O emission peak, and thus it did not significantly impact N_2_O emission flux. Previous research has shown that NosZ activity is higher under high soil pH, which may be one of the reasons for the rapid increase in nosZ gene copy numbers after crop harvest [15]. In summary, compared to traditional fertilization methods, *B. subtilis* biofertilizer reduces N_2_O production and emission by regulating the copy numbers of functional genes.

The second level involves the microbial genera in the nitrogen cycling process related to N_2_O emissions. AOB is considered an important source of soil N_2_O, and the ammonia-oxidizing bacterium Nitrosospira is the main AOB in the soil, which can produce N_2_O through denitrification. The abundance of Nitrosospira in the soil treated with *B. subtilis* biofertilizer was significantly lower than in the soil treated with chemical fertilizer, indicating that the N_2_O emissions produced by denitrification of nitrifying microorganisms were reduced. Moreover, studies have reported that when the relative abundance of Nitrospira in ammonia-oxidizing communities increases, the N_2_O emission factor from nitrification decreases exponentially, which is consistent with our results [26]. It has been reported that Pedobacter only carries atypical nosZ genes and lacks any other denitrification genes, meaning that this genus is limited to N_2_O reduction [27]. The application of chemical fertilizers significantly increased the abundance of Pedobacter, suggesting that it may have promoted the reduction of N_2_O to N_2_ to some extent, although the amount of N_2_O reduced by Pedobacter was likely too low to change the overall emission flux trend. Among the genera involved in the denitrification process, the abundance of Flavobacterium and Pseudomonas was enriched in the CK soil, and there was no significant difference in Litorilinea abundance across treatments. In addition, since Pedobacter is limited to N_2_O reduction, this suggests that fertilization treatments slowed down the soil denitrification process. In summary, *B. subtilis* biofertilizer reduces N_2_O emissions by regulating the abundance of microbial genera, thereby decreasing N_2_O production from nitrification and denitrification processes.

### 4.4. Effect of B. subtilis Biofertilizer on the Microbial Community

Different treatments had a significant impact on soil microbial diversity and community composition, showing specificity at different growth stages. In the first half of the field experiment, the CBF treatment exhibited the highest species richness, which may be attributed to the synergistic effect of biofertilizer and chemical fertilizer, promoting an increase in soil microbial abundance [28]. However, after crop harvest, the BF treatment reached the highest richness, indicating that the application of biofertilizer is more beneficial for maintaining species abundance in the long term. In terms of bacterial community composition, Proteobacteria was the dominant phylum in soil and occupied a leading position in most periods, with similar results observed in soils under different environments [29]. Although Proteobacteria is the dominant phylum in most soils, its relative abundance varies greatly with changes in environmental factors. The increased relative abundance of Cyanobacteria in the CF and CBF treatments during the stable N_2_O-emission period may be associated with elevated inorganic nitrogen availability, especially under conditions of higher NO_3_^−^-N content after rainfall. This suggests that chemical fertilization altered the structure of the microbial community in a way that may be linked to nitrogen redistribution in saline-alkali soil [30]. Proteobacteria remained the dominant phylum during most stages of the experiment, which is consistent with its broad ecological adaptability and active participation in soil carbon and nitrogen cycling. Many members of this phylum are closely involved in nitrification, denitrification, and the turnover of labile substrates; therefore, shifts in its relative abundance may reflect changes in nutrient availability and nitrogen transformation intensity under different fertilization regimes. The enrichment of Oceanobacillus in the BF treatment may have particular ecological significance under saline-alkali conditions. As a halotolerant and alkaliphilic genus, Oceanobacillus is likely better adapted to high-pH and high-salinity environments than many other soil bacteria. Its enrichment may therefore indicate that BF promoted a microbial community better suited to saline-alkali stress, which could contribute to community stability, nutrient turnover, and resilience of soil ecological functions under unfavorable field conditions [31]. The enrichment of the halophilic genus Thalassobacillus in the CBF treatment may contribute to improving the adaptability and microbial stability of saline-alkali land. Furthermore, the enrichment of Flavobacterium in the CK treatment suggests that nutrient deficiencies may increase the risk of certain potential pathogens, offering a new perspective for microbial community optimization in agricultural management.

## 5. Conclusions

In conclusion, *B. subtilis* biofertilizer effectively mitigated N_2_O emissions from saline-alkali farmland while maintaining crop yield. Compared with conventional chemical fertilization, the BF treatment reduced cumulative N_2_O emissions by 39%, increased plant nitrogen uptake, and was associated with lower abundances of Bacterial-*amoA*, *hao*, and *norB* during the N_2_O emission peak. These results suggest that the mitigation effect of *B. subtilis* biofertilizer was mainly related to suppression of nitrification-associated N_2_O production. Overall, *B. subtilis* biofertilizer shows practical potential as a sustainable alternative to conventional chemical fertilization for improving nitrogen use efficiency and reducing environmental risks in saline-alkali agricultural systems.

## Figures and Tables

**Table 1:** The soil physicochemical properties during the peak period of N_2_O emission.

Treatment	pH	TSS (g·kg^−1^)	SWC (%)	SOM (g·kg^−1^)	TN (mg·kg^−1^)	NO_3_^−^-N (mg·kg^−1^)	NH_4_^+^-N (mg·kg^−1^)	NO_2_^−^-N (mg·kg^−1^)	C/N
CK	8.23 ± 0.05 a	2.84 ± 0.08 b	25.68 ± 0.61 a	5.0 ± 0.1 a	433 ± 6.6 a	12.33 ± 0.5 bc	14.81 ± 0.16 a	0.04 ± 0.005 a	35.37 ± 0.52 c
CF	8.18 ± 0.01 a	2.24 ± 0.21 a	22.43 ± 2.19 a	5.5 ± 0.1 c	753 ± 28.249 c	11.10 ± 0.47 ab	17.51 ± 0.142 b	0.10 ± 0.002 b	21.30 ± 0.76 a
CBF	8.17 ± 0.03 a	2.32 ± 0.02 a	21.86 ± 1.69 a	5.3 ± 0.1 b	531 ± 80.4 b	12.53 ± 0.39 c	24.83 ± 0.48 d	0.25 ± 0.002 d	27.21 ± 0.41 b
BF	8.18 ± 0.02 a	2.3 ± 0.16 a	22.04 ± 2.03 a	5.5 ± 0.03 c	766 ± 21.42 c	10.94 ± 0.47 a	19.73 ± 0.4 c	0.19 ± 0.002 c	21.01 ± 0.55 a

Note: CK, control; BF, 100% biofertilizer; CF, 100% chemical fertilizer; CBF, 50% chemical fertilizer and 50% biofertilizer; TSS, total soluble salts; SWC, soil water content; SOM, soil organic matter. Values in the table represent the mean ± standard error. Different letters within the same column indicate significant differences among fertilization strategies (*p* < 0.05).

**Table 2:** Diversity index of soil bacterial community.

Time	Treatment	ACE	Chao	Shannon	Simpson	Coverage	Sobs
6.24	-	2672	2637	6.92	0.003	0.997	2626
7.11	CK	2705	2670	6.89	0.003	0.997	2659
	BF	2569	2553	7.08	0.002	0.998	2546
	CF	2535	2511	6.63	0.009	0.998	2504
	CBF	2756	2726	7.1	0.002	0.997	2709
8.22	CK	2517	2497	7.04	0.002	0.998	2493
	BF	2213	2188	6.21	0.012	0.998	2177
	CF	2280	2263	6.27	0.02	0.998	2259
	CBF	2550	2536	6.99	0.002	0.999	2533
10.12	CK	2597	2566	6.99	0.002	0.996	2544
	BF	2674	2640	7.02	0.002	0.997	2618
	CF	2455	2425	6.68	0.006	0.997	2415
	CBF	2449	2428	6.88	0.003	0.998	2419

Note: CK (control); BF (100% biofertilizer); CF (100% chemical fertilizer); CBF (50% chemical fertilizer and 50% biofertilizer).

## Data Availability

The raw sequencing data were deposited into the SRA database with accession numbers of SRP371928 and SRP371945.

## Supplementary Materials

The following supporting information can be downloaded at https://www.mdpi.com/article/10.3390/life16040635/s1, Figure S1. Linear regression analysis of N_2_O emission flux with nitrogen cycle functional genes. Different balls represent different samples; Figure S2. LEfSe identified the size of differentiation between different fertilizer treatments at different period of N_2_O emission with a threshold value of 2. CK (control); BF (100% biofertilizer); CF (100% chemical fertilizer); CBF (50% chemical fertilizer and 50% biofertilizer); Table S1. Physicochemical properties of farmland soil before the experiment began; Table S2. The primers information in this study; Table S3. The reaction procedure of functional genes.
