# Supplementary material for: Bacillus subtilis Biofertilizer Mitigates N2O Emissions from Saline-Alkali Farmland
Source: Life (Basel). 2026 Apr 9;16(4):635. doi: 10.3390/life16040635 (PMC13117370; doi:10.3390/life16040635)
Supplement: Supplementary file 1 [file life-16-00635-s001.zip › life-4177406-supplementary.pdf]

B. subtilis biofertilizer mitigates N_2O emissions from saline-alkali farmland and its potential mechanisms

Rui Li[a,b,c,*], Xingjie Lin[a], Yu miao[a], Chi Zhang[a], Fangze Li[a], Ge Zhang[a], Qiwei Sun[a,], Tianci Hua[a], Qiwei Sun[a], Jiachen Wang[b,c,*]

[a] BGRIMM Technology Group, Institute Environment Engineering, Beijing 100160, China

b Research Center for Eco-Environmental Sciences, Chinese Academy of Sciences, Beijing 100085, China.

c University of Chinese Academy of Sciences, Beijing 100049, China.

*Corresponding author: Building 23, Zone 18, Headquarters Base, 188 West Nansihuan Road, Fengtai District, Beijing 100160, China.

E-mail address: lirui18@mails.ucas.edu.cn (R. Li)

Table S1. Physicochemical properties of farmland soil before the experiment began

Treatment	pH	TSS ($mg \cdot kg^{-1}$)	SOM ($g \cdot kg^{-1}$)	EC ($ds \cdot m^{-1}$)	SWC ($g \cdot kg^{-1}$)	TN ($mg \cdot kg^{-1}$)	NO_3^--N ($mg \cdot kg^{-1}$)	NO_2^--N ($mg \cdot kg^{-1}$)	NH_4^+-N ($mg \cdot kg^{-1}$)
CK	8.102	15.627	4.04	8.102	103	473.67	17.47	0.13	14.956
BF	8.204	15.278	3.92	8.204	77.1	614.00	20.03	0.05	13.885
CF	8.153	15.218	4.13	8.153	87.9	582.00	17.35	0.05	13.606
CBF	8.225	15.802	4.08	8.225	102	611.00	23.35	0.038	13.025

Table S2. The primers information in this study

Name	Sequence (5'-3')	Types	PCR Products(bp)	Reference
Bacterial-*amoA*	GGGGTTTCTACTGGTGGT	Forward Primer	500	(Sun et al., 2020)
	CCCCTCKGSAAAGCCTTCTTC	Reverse Primer		
nosZ	CGCRACGGCAASAAGGTSMSSGT	Forward Primer	267	(Sun et al., 2020)
	CAKRTGCAKSGCRTGGCAGAA	Reverse Primer		
hao	GTMGGHTGYATYGACTGYCAC	Forward Primer	805	(Wang et al., 2024)
	GRCGRTTGGTBKTYTGDCC	Reverse Primer		
norB	GACAAGNNNTACTGGTGGT	Forward Primer	389	(Jung et al., 2012)
	GAANCCCCANACNCCNGC	Reverse Primer		

Table S3. The reaction procedure of functional genes

Project	Temperature	Time	Cycle Numbers
Pre-denaturation	95℃	5min	1
Denaturation	95℃	15sec	
Annealing	60℃	15sec	45
Extension	72℃	35sec	
	95℃	15sec	
Melting curve collection	65℃	60sec	1
	95℃	30sec	
	95℃	15sec	

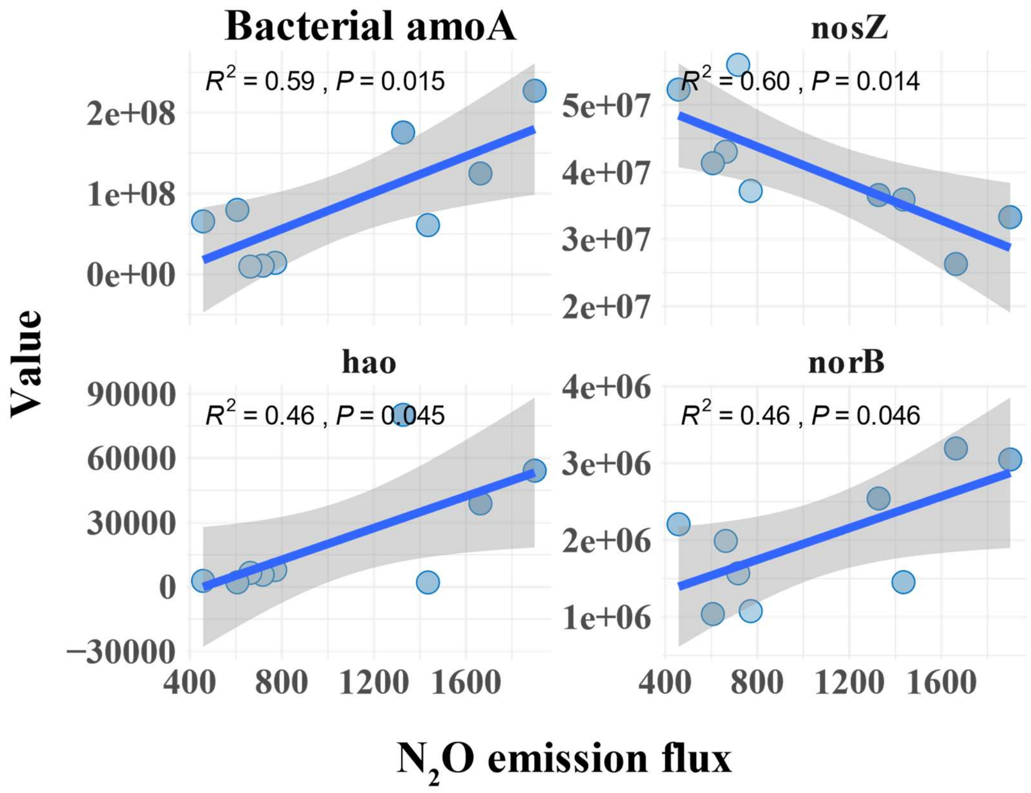

Figure S1. Linear regression analysis of N_2O emission flux with nitrogen cycle functional genes. Different balls represent different samples.

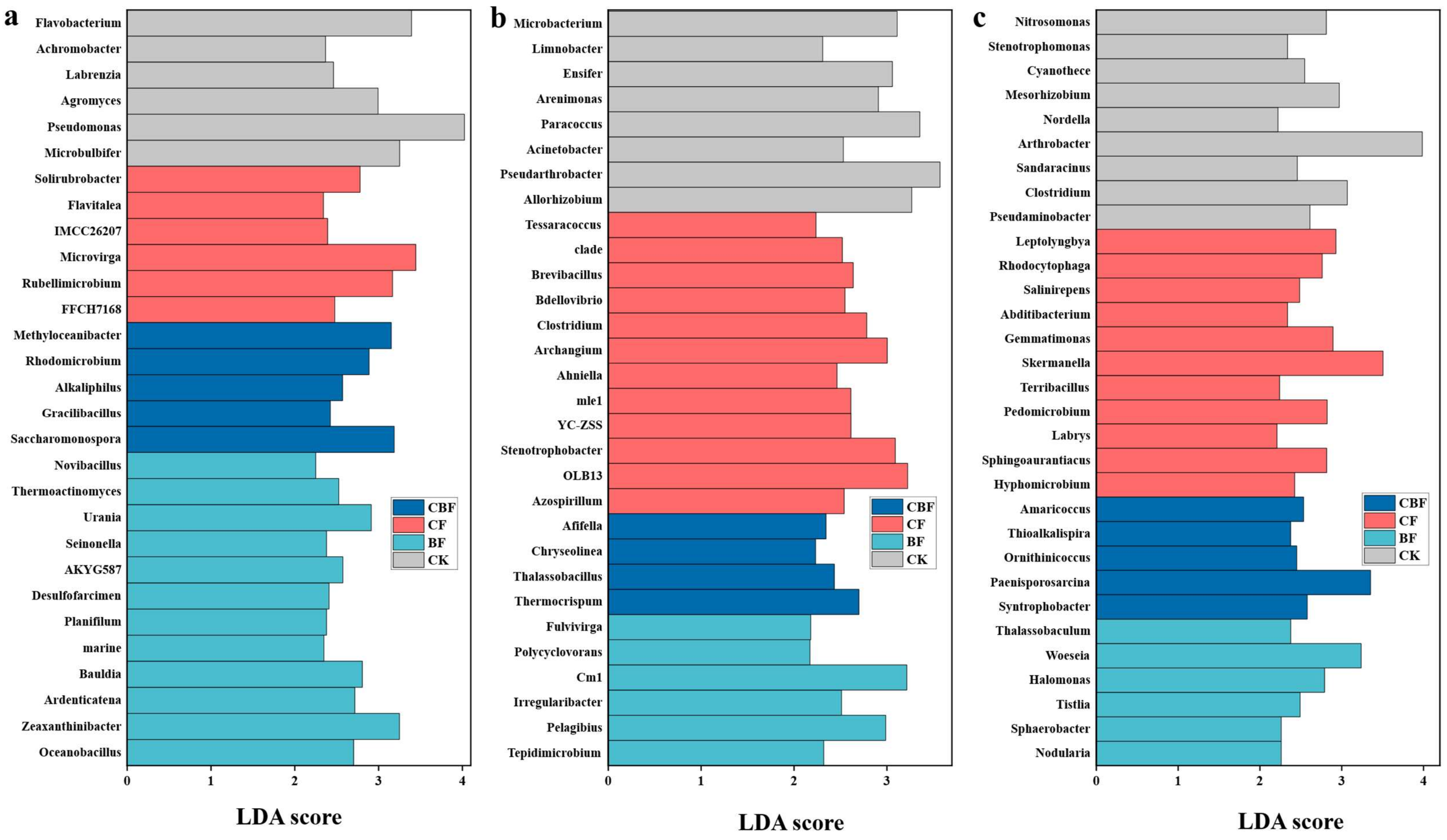

Figure S2. LEfSe identified the size of differentiation between different fertilizer treatments at different period of N_2O emission with a threshold value of 2. CK (control); BF (100% biofertilizer); CF (100% chemical fertilizer); CBF (50% chemical fertilizer and 50% biofertilizer).
